# Genome sequence of seven human herpes simplex virus 2 (HSV2) clinical isolates from Finland

**DOI:** 10.1128/mra.01208-25

**Published:** 2026-05-12

**Authors:** Christopher D. Bowen, Daniel W. Renner, Henrik Paavilainen, Tytti Vuorinen, Veijo Hukkanen, Moriah L. Szpara

**Affiliations:** 1 Department of Biology, Pennsylvania State University 8082, University Park, Pennsylvania, USA; 2 Center for Infectious Disease Dynamics, Huck Institutes of the Life Sciences, Pennsylvania State University 311285 , University Park, Pennsylvania, USA; 3 Institute of Biomedicine, University of Turku 8058, Turku, Finland; 4 Department of Clinical Virology, Turku University Hospital 1041, Turku, Finland; 5 Department of Biochemistry and Molecular Biology, Pennsylvania State University 8082, University Park, Pennsylvania, USA; Katholieke Universiteit Leuven, Leuven, Belgium

**Keywords:** herpes simplex virus 2, genomes, human herpesviruses, clinical microbiology

## Abstract

We sequenced seven herpes simplex virus 2 (HSV2) clinical isolates circulating between 2000 and 2014 in Finland. Few HSV2 virus isolates have been sequenced from Europe, particularly from Nordic countries. These sequences expand our knowledge of the global sequence diversity of HSV2 sequences and help elucidate total variation between geographic regions.

## ANNOUNCEMENT

Herpes simplex virus 2 (HSV2; *Simplexvirus humanalpha2*, subfamily *Alphaherpesvirinae*, family *Herpesviridae*) is a double-stranded DNA virus and the causative agent of recurrent genital lesions. Seven isolates were obtained from anonymous clinical diagnostic samples of herpes lesions collected between 2000 and 2014 (Table 1) (1). Approval for the study of anonymous HSV isolates was provided by the Hospital District of South West Finland, currently designated as the Wellbeing Services County of Southwest Finland (permit number J10/17). An immunoperoxidase rapid culture assay (2) was used to type the viruses as HSV2, with confirmation by a type-specific HSV-DNA-PCR (3). Viruses were initially propagated on Vero cells (ATCC). For viral genomic DNA isolation, viral stock collected from the first or second passage on Vero cells was used to infect ~1 × 10^8^ HaCaT cells (Department of Dentistry, University of Turku [4]), and the infection was allowed to proceed to completion at 35°C (1–3 days).

**TABLE 1:** Sequence data for seven HSV2 genomes from Finland

HSV2 isolate name	Genome assembly length (bp)	# of HSV-specific paired reads	Average coverage depth	GenBank accession ID	SRA accession ID
HSV2-H1227	152,782	3,764,008	1,599×	KY922721	SRX30815609
HSV2-H1229	154,136	3,119,962	1,195×	KY922722	SRX30815610
HSV2-H12211	155,111	3,605,960	1,168×	KY922725	SRX30815611
HSV2-H12212	155,788	2,866,398	1,039×	KY922726	SRX30815612
HSV2-H1226	152,981	2,864,842	1,032×	KY922720	SRX30815608
HSV2-H1526	154,678	1,375,572	532×	KY922724	SRX30815614
HSV2-H1421	148,702	196,780	35×	KY922723	SRX30815613

Viral genomic DNA was isolated as previously described (5, 6), with Freon extraction to disrupt cellular membranes, density gradient separation of capsids, and lysis with detergent and proteinase K to free viral nucleocapsid DNA. Viral DNA was extracted with phenol-chloroform, ethanol-precipitated, and quantitated by Qubit (Thermo Fisher). Input DNA (200 ng) was quantified by Qubit, sheared using a Covaris M220, and prepared for sequencing using the TruSeq Nano DNA Low Throughput Sample Preparation Guide (Illumina). Sequencing libraries were quality-controlled using Qubit, Bioanalyzer (Agilent Technologies), and qPCR (KAPA Biosystems). Libraries were loaded on an Illumina MiSeq for 2× 300 bp paired-end sequencing.

Viral genomes were assembled using our published bioinformatics workflow (installation and instructions at http://virga.readthedocs.org/ [7]). The VirGA-specific scripts maf_net.py and compare_genomes.py are archived at https://bitbucket.org/szparalab/virga/src/master/ (7). Quality control utilized FastX-Toolkit and Trimmomatic (default parameters) for adapter trimming and removal of sequencing artifacts and low-quality bases (8, 9). We used Bowtie 2 to remove any DNA sequences likely derived from HaCaT host cell DNA (using GCF_000001405.13 as a proxy) (7, 10). *De novo* assembly utilized SSAKE v3.8 (11), Celera 8.1 (12), and GapFiller 1.10 (insert_deviation 0.2) (13), followed by Mugsy 2.3 (14) and the VirGA script maf_net.py (7) to create a consensus genome for each strain from the *de novo* assembled contigs. Homology-based transfer of annotation from HSV2 strain HG52 (NC_001798 [15]) to each new consensus genome utilized the VirGA script compare_genomes.py (7). The length, coverage depth, and GenBank accession IDs for these genomes are listed in Table 1. HSV2 isolate H1421 had lower sequence coverage as a result of higher host DNA contamination, leading to a partial genome assembly with incomplete termini (Table 1).

**Fig 1:**
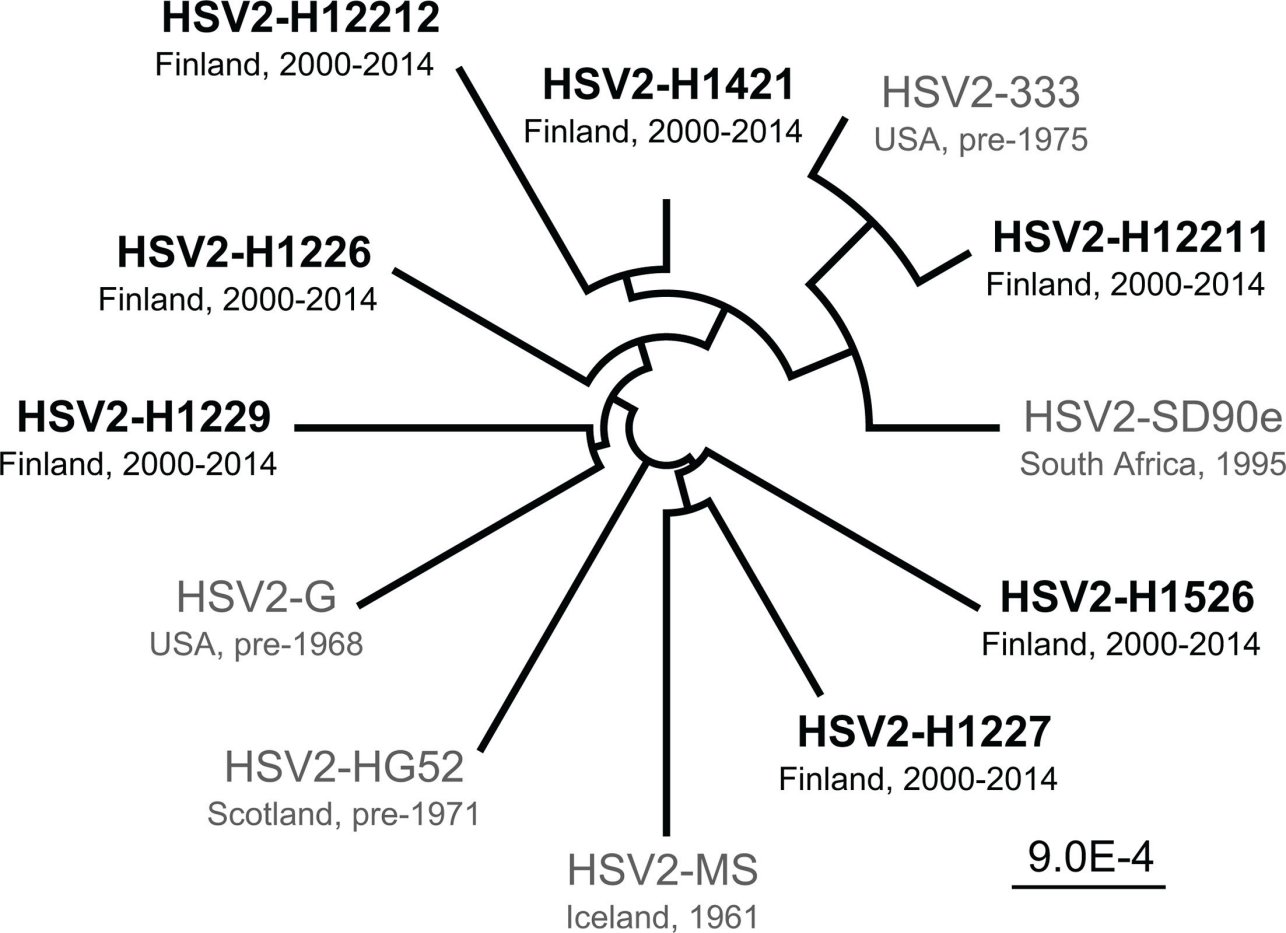
Genetic relatedness of Finnish HSV2 genomes. A neighbor-joining tree built based on a whole-genome alignment of seven Finnish HSV2 strains (bold) vs previously characterized HSV2 comparison strains HG52 (NC_001798 [15]), MS (MK855052 [18]), G (OM370995 [17]), SD90e (KF781518 [21]), and 333 (LS480640 [22]). We used MAFFT (7.490, FFT-NS-2 with default parameters [23]) to create a whole-genome alignment. A tree generated from this full-genome alignment using the neighbor-joining method (Jukes-Cantor model, Geneious Prime 2022.2.1) illustrated their overall relatedness. These seven Finnish and five comparison HSV2 genomes shared 95.1% pairwise nucleotide sequence identity (Geneious Prime 2022.2.1).

The G + C content for these viral genomes ranged from 69.1% to 70.3%. Their overall relatedness to classic HSV2 strains is shown in Fig. 1. These genome sequences have already been proven useful in reconstructing the evolutionary history of HSV2 vs the distantly related species HSV1 (16) for comparing sequence divergence in HSV2 (17, 18) and for research on new antiviral therapeutics and diagnostic approaches (19, 20).

## Data Availability

All HSV2 clinical isolate genomes have been deposited at GenBank (KY922720-KY922726), and the raw reads have been submitted to the SRA (SRX30815609, SRX30815610, SRX30815611, SRX30815612, SRX30815608, SRX30815614, SRX30815613).
